# Supplementary material for: Chromothripsis during telomere crisis is independent of NHEJ, and consistent with a replicative origin
Source: Genome Res. 2019 May;29(5):737–49. doi: 10.1101/gr.240705.118 (PMC6499312; doi:10.1101/gr.240705.118)
Supplement: Supplemental Material [file supp_gr.240705.118_Supplemental_file_1.zip › contigs/annotated_contigs/DB46/contig.2.DB46_length_545_mean_cov_3.57798165138.docx]

**DB46_length_545_mean_cov_3.57798165138**

```
TCTGACTCCTTGTGGCCTGATGAAGTCTAAGGGTTTCAGAATAAAAGGCATGCAAACTGCTACAGATCTAGCAAGCCAATATGAAGAAA
 >chr4:112620023-112620345 + E=3e-182 p=0e+00
TAAACATTCAGCACTGAAATTCTTCACATATTAATTGATCAAACGATGCATGTAGTATCAGTGCTTGAGTACACCATCATGAAGTTCAG

TACTCCCAATTTGGAACAAATGAAGGGTAACTTTTAGTCATTATTAAATTTATTTATGGAAAAAACTAAGAAAATTCTGATTAAAATTA

GTAGAATACAAAACAATATAATAAAAAAGCAAGGGAAGCAGTGGAATTTACCTTG|GAAATGTCAA|GGGTTTGAAATTTCACTTAAAA
                                                                  >chr4:112621562-11262
AGATGACATTTTAGGGCTATTTATTTACTTTTTACTATAAAAATAACAAAGGGCCATGTTGGGAAATTACAAAACAATAAAAGACAAAA
1775 + E=2e-116
TCCCTATCATCTACAAGCTAGTGATTTTAACTTATATAGTGAGAAGACATATATAATAACTCTTGAAATATAAATGAAATAACACCTCA

TGTCATCACAGTG
```
